# Association of microRNA-34a rs2666433 (A/G) Variant with Systemic Lupus Erythematosus in Female Patients: A Case-Control Study

**DOI:** 10.3390/jcm10215095

**Published:** 2021-10-30

**Authors:** Nesreen M. Ismail, Eman A. Toraih, Mai H. S. Mohammad, Eida M. Alshammari, Manal S. Fawzy

**Affiliations:** 1 Department of Rheumatology and Rehabilitation, Faculty of Medicine, Suez Canal University, Ismailia 41522, Egypt; tasneemge@yahoo.com; 2 Department of Surgery, Tulane University School of Medicine, New Orleans, LA 70112, USA; 3 Genetics Unit, Department of Histology and Cell Biology, Suez Canal University, Ismailia 41522, Egypt; 4 Department of Clinical Pathology, Faculty of Medicine, Suez Canal University, Ismailia 41522, Egypt; maii81hs@hotmail.com; 5 Department of Chemistry, College of Science, University of Ha’il, Ha’il 2440, Saudi Arabia; eida.alshammari@uoh.edu.sa; 6 Department of Medical Biochemistry and Molecular Biology, Faculty of Medicine, Suez Canal University, Ismailia 41522, Egypt; 7 Department of Biochemistry, Faculty of Medicine, Northern Border University, Arar 1321, Saudi Arabia

**Keywords:** MIR34A, lupus nephritis, single nucleotide polymorphism, SLE

## Abstract

Several microRNAs (miRNAs) are associated with autoimmune disease susceptibility and phenotype, including systemic lupus erythematosus (SLE). We aimed to explore for the first time the role of the miRNA-34a gene (MIR34A) rs2666433A > G variant in SLE risk and severity. A total of 163 adult patients with SLE and matched controls were recruited. Real-Time allelic discrimination PCR was applied for genotyping. Correlation with disease activity and clinic-laboratory data was done. The rs2666433 variant conferred protection against SLE development under heterozygous [A/G vs. G/G; OR = 0.57, 95%CI = 0.34–0.95], homozygous [A/A vs. G/G; OR = 0.52, 95%CI = 0.29–0.94], dominant [A/G + A/A vs. GG; OR = 0.55, 95%CI = 0.35–0.88], and log-additive [OR = 0.71, 95%CI = 0.53–0.95] models. Data stratification by sex revealed a significant association with SLE development in female participants under heterozygous/homozygous models (*p*-interaction = 0.004). There was no clear demarcation between SLE patients carrying different genotypes regarding the disease activity index or patients stratified according to lupus nephritis. Enrichment analysis confirmed the implication of MIR34A in the SLE pathway by targeting several genes related to SLE etiopathology. In conclusion, although the MIR34A rs2666433 variant conferred protection against developing SLE disease in the study population, it showed no association with disease activity. Replication studies in other populations are warranted.

## 1. Introduction

The systemic lupus erythematosus (SLE) (OMIM: 152700) is a complex autoimmune disease characterized by a loss of tolerance against nuclear autoantigens and complex dysfunction of innate and adaptive immunity [1]. The worldwide overall incidence rates of SLE range from 1 to 10 per 100,000 person-years, affecting predominantly females (the female/male ratio is 9:1) of a reproductive age [2]. The major pathogenic mechanisms of SLE include an inappropriate immune response to the nucleic acid-containing cellular particles, which impact different organ systems [3,4]. Clinical and epidemiological studies suggest genetic factors, in addition to environmental insults, play an important role in SLE pathogenesis [5,6,7,8].

MicroRNAs are small non-coding RNAs that predominantly bind to the 3′-untranslated region (3′-UTR) of target messenger RNAs (mRNAs) and then promote mRNAs degradation or inhibit translation [9]. Emerging studies have shown that they play important roles in various immunological and autoimmune disorders, including SLE [10,11,12].

The miR-34a gene (MIR34A), located at the chromosome 1p36 locus, is an essential modulator of the immune system [13,14]. It is widely expressed in immune cells, including B cells and T cells, regulating their function, development, and survival [14]. By targeting the Forkhead “Foxp1” transcription factor, miR-34a could inhibit B cell development at the “pro-B cell” to “pre-B cell” transition, leading to a decline in mature B cells [13]. Furthermore, it was implicated with other microRNAs in modulating the “T cell responses” process [14]. For example, miR-34a can directly target the diacylglycerol kinase zeta, and in turn, through this signal pathway, lead to enhance T cell activity [15]. Moreover, targeting five members of the protein kinase C family, miR-34a can regulate T cell migration and control cell signaling via the immunological synapses downstream of the “T cell receptors” [16]. By modulating the nuclear factor kappa B signaling in T cells, miR-34a can regulate T cell functions and control several aspects of innate/adaptive immune functions [17].

The deregulated expression of miR-34a was observed in several autoimmune disorders like multiple sclerosis [18] and rheumatoid arthritis (RA) [19], among others [14]. A recent study by Xie and colleagues reported that miR-34a derived from peripheral blood mononuclear cells of SLE patients could play a putative role in disease activity, and its gene expression levels were directly correlated with several disease indices, such as “erythrocyte sedimentation rate, anti-streptolysin antibody, rheumatoid factor, and C-reactive protein” [20]. Furthermore, by targeting Foxp3, miR-34a could limit Tregs differentiation with subsequent Tregs/Th17 cells imbalance and immune tolerance breakdown [20,21]. This suggests that miR-34a could be a potential biomarker or a new potential target for SLE disease.

Single nucleotide polymorphisms (SNPs) are ubiquitous genetic variations at certain nucleotide positions of the genome, implicated in several human disorders, including immunological diseases [22]. Further, miRNAs expression can be affected by SNPs locating in their coding genes with a subsequent change in several biological functions [23]. Earlier studies have shown that individuals’ susceptibility to human diseases may be modified by SNPs of the MIR34A gene, such as type 2 diabetes [24], ischemic stroke [25], and colon cancer [26], among others. However, the association between MIR34A variants and SLE susceptibility has not been reported before. In this sense, a common SNP in MIR34A (rs2666433: A > G) was selected based on location, minor allele frequency, and previous supporting reports. In the current case-control study, the authors aimed to investigate the association of this specified variant with SLE susceptibility and severity. The identification and characterization of such associations may highlight this variant’s role in disease susceptibility and help develop novel genetic risk stratification for targeted screening and management in the near future.

## 2. Materials and Methods

### 2.1. Study Subjects

A total of 326 adult participants (163 SLE patients and 163 controls) were recruited in this case-control study. The consecutive SLE cases were enrolled from the “Rheumatology and Nephrology Departments at the Suez Canal University (SCU) Hospitals, Ismailia, Egypt”. Based on the updated SLE classification criteria specified by the 2019 European League Against Rheumatism (EULAR)/American College of Rheumatology (ACR) classification criteria for SLE [27], cases were diagnosed by experienced clinical Rheumatologist. Patients with a history of other chronic/autoimmune disorders and cancers were excluded from the study; the control group included 163 age- and sex-matched healthy blood donors attending the blood bank in the same period with no history of chronic disorders, including autoimmune diseases. Helsinki declarations were followed during work execution, and the ethical committee of the Faculty of Medicine, SCU, approved the study (approval no. 4268). Informed written consent was obtained from all participants before taking part in the study.

### 2.2. Clinical Assessment

The demographic, clinical, and laboratory data were collected from all patients. According to disease activity, patients were classified into four groups, from mild to very high activity based on the “SLE Disease Activity Index (SLEDAI) score” that classifies SLE patients into i—No activity (SLEDAI = 0), ii—Mild activity (SLEDAI = 1:5), iii—Moderate activity (SLEDAI = 6:10), iv—High activity (SLEDAI = 11:19), and v—Very high activity (SLEDAI ≥ 20) [28]. According to the ACR criteria of lupus nephritis [27], patients were divided into 93 patients with lupus nephritis and 70 SLE with no renal involvement.

### 2.3. Blood Sampling and Laboratory Evaluations

A total of five milliliters of peripheral venous blood was withdrawn from all participants under aseptic condition and divided into two aliquots; 2 mL were collected on EDTA tubes for hematological and genetic assessment, and 3 mL blood was collected on serum separator tubes, which were subjected to centrifugation to separate serum for the biochemical and immunological evaluations.

An automated hematology analyzer CELL-DYN 1700 (Abbott Diagnostics, Abbott Park, IL, USA) was applied to evaluate the complete blood count (CBC) and complemented by microscopic differential count examination. An automated biochemical analyzer Cobas c501 (Roche Diagnostics, Manheim, Germany), was applied for liver and kidney function evaluation as well as C-reactive protein (CRP) and complement proteins (C3 and C4) estimations. The advanced Westergren method was run for erythrocyte sedimentation rate (ESR) calculation. Anti-nuclear antibodies (ANA) and anti-double-stranded DNA antibodies (Anti-dsDNA) were quantified using Bio-Rad technology through indirect immunofluorescence assay (Bio-Rad Laboratories, Hercules, CA, USA). All the laboratory tests and the quality control measurements were run according to the supplier protocols and the local laboratory guidelines.

### 2.4. DNA Extraction and Purification

Genomic DNA was extracted and processed from the buffy coat of whole blood using the QIAamp DNA extraction Mini kit for blood samples (Qiagen; Catalog #: 51104) according to the manufacturer’s protocols. The concentration and purity of genomic DNA were evaluated utilizing NanoDrop™ ND-1000 spectrophotometer (NanoDrop Technologies, Inc., Wilmington, DE, USA).

### 2.5. Genotyping of MIR34A rs2666433 A > G Variant

Real-Time TaqMan allelic discrimination polymerase chain reaction (PCR) assay (C_2800266_10) was run for detection of the specified study variant, as explained in detail in our previous work (2020). Negative controls were run in each PCR experiment to ensure the absence of amplicon contamination. A StepOne Real-Time PCR System (Applied Biosystems) was programmed as follows: an initial hold for 10 min (95 °C) followed by a 40-cycle two-step PCR (denaturation for 15 s at 95 °C and annealing/extension for 1 min at 60 °C). The SDS software version 1.3.1 (Applied Biosystems, Foster City, CA, USA) was used for allelic discrimination data recall. Genotyping was performed by two persons independently blinded to case/control status. Ten percent of the randomly selected samples were re-genotyped in separate runs to exclude the possibility of false genotype calls, with a 100% concordance rate for the results.

### 2.6. Functional Role of miRNA-34a in SLE Disease

The microRNAs involved in Systemic lupus erythematosus|hsa05322 pathway were determined from mirPath v.3, a microRNA pathway analysis webserver (DIANA TOOLS-mirPath v.3 (grnet.gr)); hsa-miR-34a-5p was the second after hsa-miR-16–5p that are highly enriched in the SLE pathway. Next, microRNA gene targets were identified from TarBase v7.0 (DIANA TOOLS-TarBase v7.0 (Athena-innovation.gr)). Their gene ontology and function were explored in STRING v11.0 (STRING interaction network (string-db.org)) (last accessed on 23 May 2021).

Validation of the role of miR-34a in SLE was screened in high-throughput experiments stored in online data repositories. Data were retrieved for similar experiments on SLE from the Gene Expression Omnibus (GEO) (Home-GEO-NCBI (nih.gov)) (last accessed on 23 May 2021) with microRNA seq analysis. Two datasets were available [GSE80183 and GSE72509], and raw data were analyzed using GEO RNA-seq Experiments Interactive Navigator (GREIN (ilincs.org)) (last accessed on 23 May 2021). and the comprehensive network visual analytics platform for gene expression analysis NetworkAnalyst (www.networkanalyst.ca) (last accessed on 23 May 2021). In the first experiment, 117 RNA-seq of SLE whole blood and healthy controls and patients were stratified according to their autoantibody status. In the second experiment, 12 SLE patients were segregated into three groups based on the presence of autoantibodies against (i) dsDNA only (ii) ENA (extractable nuclear antigens) only, or (iii) both compared to 4 control samples.

### 2.7. Selection of the Study Genetic Variant of MIR34A Gene

MIR34A gene encodes for a single primary transcript with one exon that encloses 32 variant alleles. However, they were very rare (<0.001). In dbSNP version 135, we identified a common SNP rs2666433 caused due to point mutation substituting A with G. The minor allele frequency (MAF) was 0.259 (according to 1000Genome project), 0.191 (according to TOPMED project), and 0.30 (according to HapMap). The rs2666433 polymorphism is located at 1:9213177 (chromosome 1p36.22) 2KB upstream to the MIR34A gene and overlaps the first intron of the MIR34A host gene (MIR34AHG) (position 28889 of 30171: −1283 upstream to the splicing site). Despite being predicted to be a benign variant, it was previously reported to be associated with human diseases [25,26].

### 2.8. Statistical Analysis

Statistical analysis was performed by GraphPad Prism v9.0 and Statistical Package for Social Science (version 27.0). The Shapiro–Wilk test was applied to assess the normality of continuous variables, then analyzed by the Kruskal-Wallis or Mann–Whitney *U* tests (if non-parametric) or the One-Way ANOVA or Student’s *t*-test (if parametric). Genotype and allele frequencies were estimated as previously described, and SNPstats software was applied [29]. Genotypes and alleles distribution of rs2666433 in different populations were compared from the Ensemble database (ensemble.org) (last accessed on 23 May 2021). Hardy–Weinberg equilibrium (HWE) testing and categorical variables comparison were preceded by a two-sided Chi-Square test. Logistic regression was used for evaluating the association between miR-34a polymorphisms and SLE disease risk, and odds ratio (OR), 95% confidence interval (CI), and *p*-values were adjusted by age and sex; *p* < 0.05 indicated statistical significance. The principal component analysis was plotted using R packages. The power calculation was performed by the G*Power version 3.1.9.2. Under the parameters of α error probability = 0.05, total sample size (326; each case/control subgroup = 163), and calculated effect size = 0.288, the study has 99% statistical power to identify a convincing association between the rs2666433 variant and SLE risk.

## 3. Results

### 3.1. Characteristics of the Study Population

This case-control study included 163 SLE patients (147 females and 16 males) and 163 controls (148 females and 15 males). Their mean age was 35.6 ± 9.6 years for patients and 35.8 ± 9.9 years for controls. The characteristics of the study population are demonstrated in Table 1. Females represented 90% of the SLE population. Positive family history (history of rheumatic diseases and/or autoimmune diseases as SLE, RA, autoimmune thyroid disease, diabetes mellitus type 1, inflammatory bowel disease, and psoriasis) was identified in 35% of patients. Almost 49% of patients presented with arthritis/arthralgia symptoms, CNS manifestations (25%), and peripheral neuropathy (42%). According to the SLEDAI score, patients were divided into four groups: 7.4% Grade 1, 30% Grade 2, 34% Grade 3, and 23% Grade 4.

Comparison between patients with different disease activities is illustrated in Table 2. As expected, oral ulcers presented in 44% of patients within grade 4 (*p* = 0.043), while lupus nephritis was more prevalent in patients within grades 3 and 4 (60% and 82%, respectively) (*p* < 0.001). In addition, significant differences regarding casts in urine and proteinuria were evident (*p* > 0.001).

### 3.2. Role of miRNA-34a in SLE Disease

**Figure 1:**
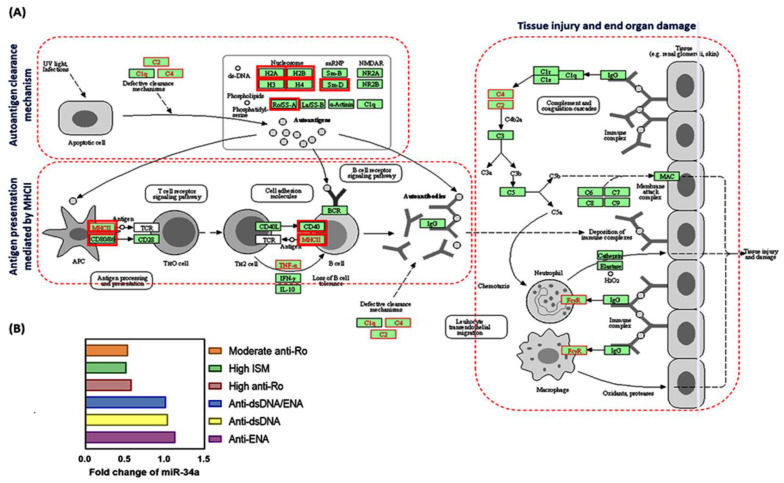
SLE is an autoimmune disease characterized by augmented self-antigen specific auto-antibodied production that contribute to a pleotropic clinical presentation. (**A**) MicroRNA-34a is significantly enriched in the SLE pathway. Gene targets of miR-34a were labeled in red bold-colored boxes in the SLE KEGG pathway (hsa05322) [30]. MiR-34a target genes code for different histone family members (i.e., H2A, H2B, H3, and H4) that are implicated in tolerance mechanism/autoantigen clearance and the “major histocompatibility complex class II (MHCII)”, which plays an essential role in antigen presentation [31]. All the stages of antigen processing and autoantibodies generation lead to tissue injury and end-organ damage (left panel) [32]. More detail for gene names and functions are provided in Table S1. Data source: Diana Lab tools (DIANA TOOLS-Reverse Mirpath (grnet.gr) (last accessed on 25 May 2021). (**B**) The expression level of the MIR34A gene in SLE patients in independent cohorts from Gene Expression Omnibus datasets (GSE80183 and GSE72509), Anti-Ro: a type of RNA-binding proteins; ISM: Interferon Signature Metric; dsDNA: double-stranded DNA; ENA: extractable nuclear antigens (RNAassociated proteins) [33,34].

As depicted in Figure 1A, miR-34a-5p has 27 experimentally validated gene targets in SLE KEGG pathway (hsa05322|Adj *p*-value = 2.4 × 10^−7^, including several histone variants, RNA-binding proteins, and immune response-related genes (Table S1). Validation of miR-34a expression in two independent SLE cohorts from online GEO datasets revealed microRNA upregulation in patients compared to controls (Figure 1B). In GSE80183, miR-34a was upregulated in patients with anti-dsDNA (FC = 1.04, *p* = 0.017), anti-ENA (FC = 1.133, *p* = 0.015), and both anti-dsDNA and anti-ENA (FC = 1.016, *p* = 0.039) in comparison with healthy individuals. In GSE72509, relative expression was 0.579 (*p* = 11.7 × 10^−7^) and 0.538 (*p* = 18.6 × 10^−6^) in patients with high anti-Ro and moderate anti- Ro, and 0.514 (*p* = 11.8 × 10^−8^) in patients with high Interferon Signature Metric (ISM) compared to controls.

### 3.3. MIR34A rs2666433 Genotype and Allelic Frequencies

**Figure 2:**
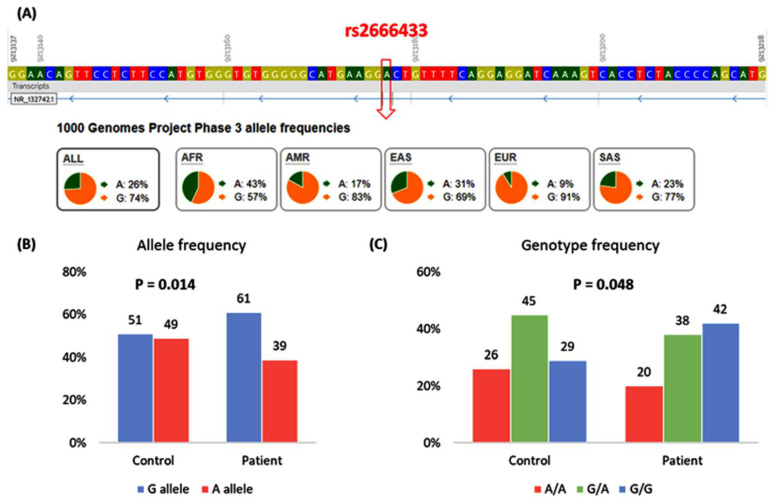
Genotype and allele frequencies of MIR34A rs2666433 (A/G) polymorphism. (**A**) Genomic location and allele frequencies of rs2666433 variant. Data source: (Ensembl.org) (last accessed on 25 May 2021). (**B**) Allele frequency in the current study. (**C**) Genotype frequency in the current study. Values are shown as a percentage. A Chi-square test was used. *p*-value < 0.05 was considered as statistically significant. The *p*-value for Hardy–Weinberg equilibrium is 0.21.

The HWE among controls was in line with observed equilibrium (*p* = 0.21). The A allele of the rs2666433 variant was 0.39 among patients with SLE compared to 0.49 among controls, while the G allele frequency was 0.61 among patients vs. 0.51 in healthy controls (*p* = 0.014) (Figure 2B). The most predominant genotype among patients with SLE was G/G genotype (42%), while the most frequent genotype among controls was the G/A genotype (45%) (Figure 2C).

### 3.4. Association of MIR34A rs2666433 Variant with SLE Development

Upon adjusting the covariates; age and sex, the MIR34A rs2666433 polymorphism conferred a protection against developing SLE under several genetic models, including heterozygous [A/G versus G/G; OR = 0.57, 95%CI =0.34–0.95], homozygous [A/A vs. G/G; OR = 0.52, 95%CI = 0.29–0.94], dominant [A/G + A/A vs. GG; OR = 0.55, 95%CI = 0.35–0.88], and log-additive [OR = 0.71, 95%CI = 0.53–0.95] models (Table 3). When genotyping data stratified by sex, the study variant showed significant association with SLE development in female participants compared to males under heterozygous model (OR = 0.45, 95%CI = 0.26–0.77) and homozygous model (OR = 0.39, 95%CI = 0.21–0.74) (*p*-interaction was 0.004) (Table 4).

### 3.5. Association of MIR34A rs2666433 Variant with Clinic-Laboratory Variables

Table 5 indicates that among A/A carriers there were a higher proportion of photosensitive patients (*p* = 0.002), experiencing weight loss (*p* = 0.011), anemia (*p* = 0.005), lymphopenia (*p* = 0.048), and raising blood urea (*p* = 0.034), in comparison to A/G and G/G carriers.

### 3.6. Impact of MIR34A rs2666433 Variant on the Disease Activity Index

**Figure 3:**
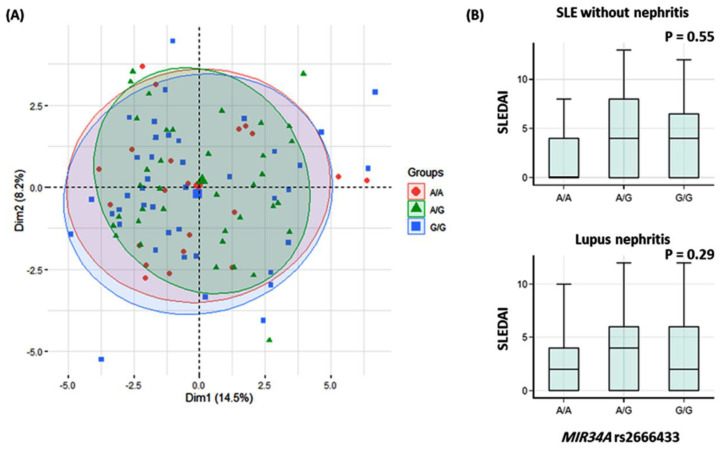
Impact of MIR34A rs2666433 (A/G) variant on disease activity index. (**A**) The principal component analysis for data exploration showed no clear demarcation between patients with different genotypes. (**B**) Box plots in SLE with and without nephritis show no significant difference in SLE disease activity index (SLEDAI).

The principal component analysis for data exploration showed no clear demarcation between SLE patients carrying different genotypes regarding the disease activity index (Figure 3A). Moreover, the study variant genotypes showed no significant association with SLEDAI upon stratifying patients according to the presence or absence of lupus nephritis (*p* = 0.29 and = 0.55, respectively) (Figure 3B).

## 4. Discussion

Accumulating evidence indicates that a “dose-dependent combination” of susceptibility genes, estrogenic hormones, immunological and environmental factors are involved in lupus etiopathology [35,36,37]. Unraveling the genetic/epigenetic contribution to SLE pathogenesis will pave the road to personalized medicine [38].

The microRNA family of non-coding RNAs has been implicated in immune system homeostasis, and its genetic variants and gene signature deregulation are associated with several immunological disorders, including SLE [32,39,40,41,42]. The present study identified for the first time that MIR34A rs2666433 A/A and A/G genotypes are less likely to develop SLE in the study population. On searching the national library of medicine (https://www.ncbi.nlm.nih.gov/snp/?term=rs2666433) (last accessed on 23 May 2021), there are only two rs2666433-related studies; one relates this specified variant with ischemic stroke in the Chinese population [25], and another publication explored the impact of this variant on CRC risk [26]. Sun and colleagues reported that this variant might impact the binding of several transcription factors to promoter elements of this gene [24] that results in a change of the type of activated/suppressed gene targets. Indeed, Wei and colleagues reported that “patients with ischemic stroke carrying A/A genotype had a higher level of transcript levels than carriers of G/G and G/A genotypes” [25], suggesting that this variant may impact gene expression level. The exact molecular mechanism(s) by which the rs2666433 variant implicated in SLE pathogenesis will need further functional analyses to unleash the relation of this SNP to SLE risk.

On searching “HaploReg V4.1: an online tool for exploring annotations of the non-coding genome (https://pubs.broadinstitute.org/mammals/haploreg/haploreg.php) (last accessed on 27 May 2021) [43] to predict the impact of rs2666433 SNP, we found that it is in linkage disequilibrium (r^2^ ≥ 0.8) with other variants present in chromosome 1 (i.e., rs34196792, rs113390912, rs34174278, and rs34619897), and can influence the Ets, peroxisome proliferator-activated receptor (PPAR), and the paired box-4 (Pax-4) DNA motifs (Table S2). Dysregulation of the specified transcriptional factors and/or their binding to these motifs was associated with autoimmune disorders in previous reports, including SLE susceptibility and pathogenesis [44,45,46]. For example, Ets1 has been reported to regulate lymphocyte/plasma cell differentiation, B cell tolerance to self-antigens, and autoantibodies/cytokine production [44], PPARγ was implicated in the regulation of the inflammatory signal initiated by CD40/CD40L activation [44], and PAX4 expression signature was identified in the differentially expressed significant probes of peripheral cells samples in patients with SLE and/or vasculitis [47].

Furthermore, our in silico analysis confirmed the implication of miR-34a in the SLE pathway (Figure 1 and Table S1) by targeting several genes coding for different histone family proteins, RNA-binding proteins, including the spliceosome small nuclear ribonucleoproteins, and several immune response-related proteins as CD86, CD40, and HLA class II histocompatibility antigen; DM alpha chain (Table S1). The functional roles of these target genes could support the significant association of the studied variant with SLE development and other phenotypic features observed in the present study.

Interestingly, when genotyping data stratified by sex, the rs2666433 variant showed significant association with SLE development in female participants compared to males under heterozygous and homozygous genetic models. Apart of the predominance of female gender among SLE participants in the present study and previous ones [8,48], estrogen has been reported to stimulate the secretion of pro-inflammatory cytokines such as “interleukin-17 and interferon-α”, which are implicated in SLE etiopathology, either through “the modification of key transcription factors in inflammation or through the regulation of miRNA expression” [49]. Several microRNAs were identified to be dysregulated in SLE under the influence of estrogen, such as miR-146a, miR-155, miR-125a, miR-181a/b, and miR-21 [49,50,51,52]. Future in vivo and in vitro studies are recommended to investigate whether miR-34a gene signature/variants in SLE could be influenced by estrogen to confirm the current finding.

Although overall analysis and stratified analysis by presence/absence of lupus nephritis demonstrated an insignificant association of the rs2666433 variant with disease severity, it showed significant association with some clinical features, including arthritis. Interestingly, Kurowska–Stolarska et al. reported that miR-34a could be an “epigenetic regulator” of the dendritic cells-mediated arthritis in patients with RA through tyrosine kinase receptor (AXL) downregulation and auto-reactive T cells activation [19]. Moreover, in vitro study has implicated miR34a in osteoarthritis synovial cell apoptosis via regulation of TGIF2 [53]. Furthermore, Zhang et al. uncovered the essential role of the long non-coding RNA “UFC1” in the osteoarthritis-associated chondrocyte survival through miR-34a sponging [54]. In addition, MiR-34a was reported to promote “Fas-mediated cartilage endplate chondrocyte apoptosis” by targeting Bcl-2 [55]. Taken together, all these studies highlight the important role of miR-34a could play in several types of arthritis, and hence the miR-34a-related variant could be associated with arthritis and other phenotypic features (such as photosensitivity and weight loss) according to the type of miR-34a target genes/molecular pathways dysregulated.

Regarding the laboratory variables, we found that among A/A carriers there were a higher proportion of patients experiencing anemia, lymphopenia and raising blood urea, in comparison to A/G and G/G carriers. As mentioned above, allele A substitution to allele G has been found to disrupt several transcriptional factor-binding DNA motifs with subsequent change in the type of deregulated genes. In this sense, the presence of double dose of allele “A” as an AA genotype might promote and impact the expression of gene set which differs from that expressed or affected by AG and/or GG genotypes.

It is worth noting that besides the current gene variant, additional contributing genetic/epigenetic and environmental factors play essential roles in SLE etiopathology. Furthermore, this study is limited by the relatively small sample size from which an association conclusion could be drawn. The exploratory nature of the study design also lacks the follow-up of patients and inability to unravel the molecular mechanism(s) by which the current variant could impact the disease. In this sense, future longitudinal confirmatory studies in large-scale cohorts, including functional experiments are required to decipher the biological significance of the MIR34A (rs266643 A > G) variant in SLE.

## 5. Conclusions

The present study revealed higher occurrence of MIR34A (rs266643) GG genotype carriers in SLE cohort of the present population rather than the AG/AA genotypes. Further large-scale studies supported by functional analyses on different ethnicities are highly recommended to explore the genotype-phenotype association details of this variant.

## Figures and Tables

**Table 1:** Baseline characteristics of SLE patients.

Characteristics		*n* = 163
Demographics		
Sex	Male	16 (9.8)
	Female	147 (90.2)
Family history	Negative	105 (64.4)
	Positive	58 (35.6)
Clinical manifestations		
Organ involvement	Malar rash	109 (66.9)
	Discoid rash	77 (47.2)
	Photosensitivity	61 (37.4)
	Hair loss	129 (79.1)
	Oral ulcer	46 (28.2)
	Arthritis	81 (49.7)
	Ecchymosis	19 (11.7)
	Fever	30 (18.4)
	Infection	26 (16)
	Dyspnea	68 (41.7)
	Chest pain	35 (21.5)
	Cough	35 (21.5)
	CNS	41 (25.2)
	Peripheral neuropathy	70 (42.9)
	Lupus nephritis	98 (60.1)
	Hematuria	56 (34.4)
	Weight loss *	76 (46.6)
Severity		
SLEDAI score	Mean ± SD	15.97 ± 9.82
	Grade 1	12 (7.4)
	Grade 2	50 (30.7)
	Grade 3	56 (34.4)
	Grade 4	45 (27.6)
Markers for severity and kidney damage	Hypocomplementemia	47 (28.8)
	Elevated inflammatory markers	67 (41.1)
	High S. creatinine	72 (44.2)
	Casts in urine	29 (17.8)
	Proteinuria	92 (56.4)
	Thrombocytopenia	5 (3.1)
Laboratory data		
Autoantibodies	Positive dsDNA	147 (90.2)
	Positive ANA titer	162 (99.4)
Biochemical tests	Hemoglobin (g/dL)	11.66 ± 2.89
	RBC (×10^6^ per mm^3^)	4.09 ± 0.74
	HCT (%)	38.18 ± 6.05
	MCV (fl)	81.42 ± 6.36
	Platelet count (×10^3^/mm^3^)	264.51 ± 77.59
	WBC (×10^3^ /uL)	6.58 ± 2.22
	Neutrophil (%)	63.30 ± 10.46
	Lymphocyte (%)	30.01 ± 9.66
	C3 (mg/dL)	95.52 ± 47.86
	C4 (mg/dL)	27.94 ± 15.62
	CRP (mg/L)	2.95 ± 2.89
	ESR 1st h	26.84 ± 13.58
	ALT (U/L)	26.61 ± 9.62
	AST (U/L)	26.50 ± 8.48
	Serum creatinine (mg/dL)	1.18 ± 1.19
	Blood urea (mg/dL)	35.11 ± 11.86

Values are shown as number (%) or mean and standard deviation. Positive family history: history of rheumatic diseases and/or autoimmune diseases as SLE, RA, autoimmune thyroid disease, diabetes mellitus type 1, inflammatory bowel disease, and psoriasis), *: “Unintentional weight loss of >5% of body weight over 6–12 months” [8]. CNS: the central nervous system; SLEDAI: Systemic Lupus Erythematosus Disease Activity index; dsDNA: Double-stranded deoxyribonucleic acid; ANA: Anti-nuclear antibody; RBC: red blood cell; HCT: hematocrit; MCV: mean cell volume; WBC: white blood cell; C3/4, complement 3/4; CRP: C—reactive protein; ALT: alanine transaminase; AST: aspartate transaminase.

**Table 2:** Characteristics of SLE patients according to their disease activity.

Characteristics		Grade 1 (*n* = 12)	Grade 2 (*n* = 50)	Grade 3 (*n* = 56)	Grade 4 (*n* = 45)	*p*-Value
Demographics						
Age, years	Median (quartiles)	27.5 (26–38)	35 (30–43.5)	36 (29.8–43.3)	36 (30.3–42.8)	0.43
Sex	Male	1 (8.3)	4 (8)	5 (8.9)	6 (13.3)	0.82
	Female	11 (91.7)	46 (92)	51 (91.1)	39 (86.7)	
Family history	Negative	6 (50)	36 (72)	36 (64.3)	27 (60)	0.43
	Positive	6 (50)	14 (28)	20 (35.7)	18 (40)	
Clinical manifestations						
Organ involvement	Malar rash	10 (83.3)	35 (70)	38 (67.9)	26 (57.8)	0.33
	Discoid rash	5 (41.7)	21 (42)	28 (50)	23 (51.1)	0.77
	Photosensitivity	5 (41.7)	18 (36)	20 (35.7)	18 (40)	0.95
	Hair loss	11 (91.7)	39 (78)	49 (87.5)	30 (66.7)	0.05
	Oral ulcer	3 (25)	11 (22)	12 (21.4)	20 (44.4)	**0.043**
	Arthritis	5 (41.7)	23 (46)	33 (58.9)	20 (44.4)	0.39
	Ecchymosis	1 (8.3)	6 (12)	10 (17.9)	2 (4.4)	0.21
	Fever	1 (8.3)	15 (30)	9 (16.1)	5 (11.1)	0.07
	Infection	2 (16.7)	8 (16)	9 (16.1)	7 (15.6)	1.00
	Dyspnea	8 (66.7)	20 (40)	22 (39.3)	18 (40)	0.34
	Chest pain	2 (16.7)	7 (14)	20 (35.7)	6 (13.3)	**0.016**
	Cough	6 (50)	8 (16)	16 (28.6)	5 (11.1)	**0.011**
	CNS	4 (33.3)	13 (26)	15 (26.8)	9 (20)	0.76
	Peripheral neuropathy	7 (58.3)	20 (40)	25 (44.6)	18 (40)	0.67
	Lupus nephritis	3 (25)	24 (48)	34 (60.7)	37 (82.2)	**<0.001**
	Hematuria	5 (41.7)	17 (34)	20 (35.7)	14 (31.1)	0.91
	Weight loss *	6 (50)	25 (50)	24 (42.9)	21 (46.7)	0.90
Severity						
Markers for severity and Kidney damge	Hypocomplementemia	3 (25)	15 (30)	13 (23.2)	16 (35.6)	0.58
	Elevated Inflammatory markers	7 (58.3)	23 (46)	21 (37.5)	16 (35.6)	0.42
	High S. creatinine	1 (8.3)	18 (36)	28 (50)	25 (55.6)	**0.013**
	Casts in urine	1 (8.3)	1 (2)	10 (17.9)	17 (37.8)	**<0.001**
	Proteinuria	1 (8.3)	21 (42)	34 (60.7)	36 (80)	**<0.001**
	Thrombocytopenia	1 (8.3)	1 (2)	1 (1.8)	2 (4.4)	0.59
Laboratory data						
Autoantibodies	Positive dsDNA	8 (66.7)	41 (82)	54 (96.4)	44 (97.8)	0.001
	Positive ANA titer	12 (100)	49 (98)	56 (100)	45 (100)	0.52
Biochemical tests	Hemoglobin (g/dL)	11.5 (10.6–13.1)	11.2 (10.4–12.1)	11.4 (10.7–12.3)	11.2 (10.4–12.9)	0.87
	RBC (×10^6^ per mm^3^)	3.9 (3.6–4.3)	4 (3.6–4.5)	4.2 (3.8–4.6)	4.1 (3.7–4.5)	0.42
	HCT (%)	39 (30.5–41)	38 (34–41)	39 (33–42)	39 (36.3–42)	0.52
	MCV (fl)	81.8 (77–89.3)	80 (76–88)	82 (80.3–87)	79.5 (77–87)	0.56
	Platelet count (×10^3^ per mm^3^)	227 (179.5–261)	248 (211.5–321)	239.5 (212–325)	268.5 (201–320)	0.50
	WBC (x10^3^ /uL)	6.4 (4.1–7.6)	6.5 (5.3–7.7)	7 (5–7.9)	6.1 (5.3–7.7)	0.62
	Neutrophil (%)	70.5 (57–71)	66 (56–71)	66 (54.8–70)	65 (59–70.8)	0.86
	Lymphocyte (%)	27 (23–35)	29 (22–35.5)	31.5 (22–39)	30 (26–34.5)	0.65
	C3 (mg/dL)	89.5 (83–120.3)	88 (50–123)	91.5 (50–123)	122 (93–138)	**0.003**
	C4 (mg/dL)	35 (27–41.5)	25 (9–37)	32.5 (8.8–42.3)	37 (23–43)	**0.028**
	CRP (mg/L)	2.9 (1.5–3.9)	2.1 (1.7–3.4)	2.8 (1.4–3.7)	2.5 (1.8–3.2)	0.81
	ESR 1st h	26 (20.8–33)	23 (16.5–36)	23 (19–33)	21 (15.3–30)	0.31
	ALT (U/L)	28.5 (22.3–30)	28 (19.5–33)	29 (19–33.5)	27.5 (17.3–33)	0.99
	AST (U/L)	25 (23.3–33.8)	28 (22–34)	27 (19.8–33)	24 (15.3–33)	0.26
	Serum creatinine (mg/dL)	1 (0.8–1.2)	1.1 (0.9–1.2)	0.9 (0.7–1.1)	1 (0.8–1.3)	**0.022**
	Blood urea (mg/dL)	36 (29–41.8)	33 (29–39)	33 (29–42)	33 (28–40)	0.80

Values are shown as number (%) or median quartiles. Chi-square and Student *t*-tests were used. Positive family history: history of rheumatic diseases and/or autoimmune diseases as SLE, RA, autoimmune thyroid disease, diabetes mellitus type 1, inflammatory bowel disease, and psoriasis), *: “Unintentional weight loss of >5% of body weight over 6–12 months” [8]. Bold values indicate statistically significant *p*-value < 0.05. CNS: central nervous system; dsDNA: Double-stranded deoxyribonucleic acid; ANA: Anti-nuclear antibody; RBC: red blood cell; HCT: hematocrit; MCV: mean cell volume; WBC: white blood cell; C3/4, complement 3/4; CRP: C—reactive protein; ESR: erythrocyte sedimentation rate; ALT: alanine transaminase; AST: aspartate transaminase.

**Table 3:** Risk of systemic lupus erythematosus by genetic association models of miR-34a rs2666433 (A/G) genotypes.

Model	Genotype	Controls (*n* = 163)	Cases (*n* = 163)	Adjusted OR (95% CI)	*p*-Value
Codominant	G/G	47 (28.8%)	68 (41.7%)	1.00	**0.041**
	A/G	73 (44.8%)	62 (38%)	**0.57 (0.34–0.95)**	
	A/A	43 (26.4%)	33 (20.2%)	**0.52 (0.29–0.94)**	
Dominant	G/G	47 (28.8%)	68 (41.7%)	1.00	**0.012**
	A/G-A/A	116 (71.2%)	95 (58.3%)	**0.55 (0.35–0.88)**	
Recessive	G/G-A/G	120 (73.6%)	130 (79.8%)	1.00	0.18
	A/A	43 (26.4%)	33 (20.2%)	0.70 (0.42–1.18)	
Over dominant	G/G-A/A	90 (55.2%)	101 (62%)	1.00	0.21
	A/G	73 (44.8%)	62 (38%)	0.75 (0.48–1.17)	
Log-additive	---	---	---	**0.71 (0.53–0.95)**	**0.02**

Values are shown as numbers (%). A chi-square test was used. OR (95% CI), odds ratio, and 95% confidence interval. Bold values indicate statistically significant *p*-value < 0.05. Adjusted covariates were age and sex.

**Table 4:** Stratified analysis by sex for genotype frequencies between cases and controls.

**SNP**	**Females**			**Males**		
	Controls *n*	Cases *n*	Adjusted OR (95% CI)	Controls *n*	Cases *n*	Adjusted OR (95% CI)
G/G	38	65	1.00	9	3	1.00
A/G	69	54	**0.45 (0.26–0.77)**	4	8	5.85 (0.99–34.62)
A/A	41	28	**0.39 (0.21–0.74)**	2	5	7.19 (0.88–58.93)

Values are shown as numbers (N). Chi-square and Fisher exact tests were applied. OR (95% CI): odds ratio and confidence interval. Bold values indicate statistically significant *p*-value < 0.05. Adjusted OR with age. *p*-interaction was 0.004.

**Table 5:** Association between miR-34a rs2666433 (A/G) genotypes and the clinic-laboratory variables.

	Total	A/A	A/G	G/G	*p*-Value
Total number	163	33	62	68	
Early onset	112	20 (60.6)	41 (66.1)	51 (75)	0.29
Female	147	28 (84.8)	54 (87.1)	65 (95.6)	0.13
Positive FH	58	7 (21.2)	31 (50)	20 (29.4)	**0.008**
Severe stage	44	10 (30.3)	15 (24.2)	19 (27.9)	0.79
Organ involvement					
Malar rash	109	18 (54.5)	41 (66.1)	50 (73.5)	0.16
Discoid rash	77	12 (36.4)	26 (41.9)	39 (57.4)	0.08
Photosensitivity	61	21 (63.6)	19 (30.6)	21 (30.9)	**0.002**
Hair loss	129	27 (81.8)	48 (77.4)	54 (79.4)	0.87
Oral ulcer	46	9 (27.3)	19 (30.6)	18 (26.5)	0.86
Arthritis	81	9 (27.3)	37 (59.7)	35 (51.5)	**0.010**
Ecchymosis	19	4 (12.1)	9 (14.5)	6 (8.8)	0.59
Fever	30	3 (9.1)	12 (19.4)	15 (22.1)	0.28
Infection	26	4 (12.1)	9 (14.5)	13 (19.1)	0.61
Dyspnea	68	18 (54.5)	27 (43.5)	23 (33.8)	0.13
Chest pain	35	7 (21.2)	15 (24.2)	13 (19.1)	0.78
Cough	35	4 (12.1)	15 (24.2)	16 (23.5)	0.34
CNS	41	12 (36.4)	12 (19.4)	17 (25)	0.19
Peripheral neuropathy	70	18 (54.5)	27 (43.5)	25 (36.8)	0.23
Hematuria	56	9 (27.3)	17 (27.4)	30 (44.1)	0.08
Renal injury	93	22 (66.7)	32 (51.6)	39 (57.4)	0.36
Weight loss *	76	23 (69.7)	24 (38.7)	29 (42.6)	**0.011**
Laboratory test					
Hemoglobin (g/dL)		10.97 ± 2.08	12.54 ± 4.03	11.08 ± 1.33	**0.005**
RBC (×10^6^/mm^3^)		3.81 ± 1.25	4.31 ± 0.62	3.94 ± 0.60	**0.004**
HCT (%)		38.21 ± 7.01	39.15 ± 5.69	37.01 ± 5.76	0.13
MCV (fl)		80.85 ± 6.20	82.63 ± 5.82	80.78 ± 6.79	0.20
Platelet count (×10^3^ per mm^3^)		292.21 ± 94.31	255.84 ± 69.54	257.40 ± 72.93	0.06
WBC (×10^3^ /uL)		6.99 ± 2.06	6.35 ± 1.94	6.51 ± 2.52	0.41
Neutrophil (%)		66.42 ± 10.49	62.35 ± 10.70	63.00 ± 9.89	0.17
Lymphocyte (%)		26.61 ± 10.45	31.87 ± 10.41	30.44 ± 9.03	**0.048**
C3 (mg/dL)		100.63 ± 56.09	94.31 ± 39.65	94.69 ± 52.05	0.81
C4 (mg/dL)		24.63 ± 16.29	28.22 ± 13.50	29.44 ± 16.64	0.33
CRP (mg/L)		2.48 ± 1.14	2.56 ± 1.27	3.43 ± 4.16	0.14
ALT (U/L)		25.09 ± 10.88	28.11 ± 9.83	25.93 ± 8.57	0.26
AST (U/L)		24.30 ± 8.60	29.02 ± 8.99	26.18 ± 8.33	**0.030**
Serum creatinine (mg/dL)		1.39 ± 1.85	1.07 ± 0.73	1.15 ± 1.11	0.44
Blood urea (mg/dL)		39.01 ± 19.71	35.40 ± 10.30	32.58 ± 6.63	**0.034**

Early onset: Age at diagnosis <40 years, severe Stage: SLEDAI score > 6. A chi-square test was used for frequency comparison. Analysis of variance (ANOVA) test is applied for data presented as mean ± SD between different subgroups. *: “Unintentional weight loss of >5% of body weight over 6–12 months” [8]. Bold values indicate statistically significant *p*-value < 0.05. Positive family history: family history of rheumatic diseases and/or autoimmune diseases as SLE, RA, autoimmune thyroid disease, diabetes mellitus type 1, inflammatory bowel disease, and psoriasis); CNS: the central nervous system; RBC: red blood cell; HCT: hematocrit; MCV: mean cell volume; WBC: white blood cell; C3/4, complement 3/4; CRP: C—reactive protein; ALT: alanine transaminase; AST: aspartate transaminase.

## Data Availability

All generated data in this study are included in the article and supplementary material.

## Supplementary Materials

The following are available online at https://www.mdpi.com/article/10.3390/jcm10215095/s1, Table S1: Gene targets for microRNA-34a-5p in the SLE KEGG pathway (hsa05322) and their structural and functional role in the cell. Table S2: Impact and linkage disequilibrium of MIR34A rs2666433 variant with other SNPs (r^2^ ≥ 0.8) predicted by HaploReg V4.1.
