# Supplementary material for: Association of microRNA-34a rs2666433 (A/G) Variant with Systemic Lupus Erythematosus in Female Patients: A Case-Control Study
Source: J Clin Med. 2021 Oct 30;10(21):5095. doi: 10.3390/jcm10215095 (PMC8584584; doi:10.3390/jcm10215095)
Supplement: Supplementary file 1 [file jcm-10-05095-s001.zip › jcm-1376108-supplementary.pdf]

Table S1. Gene targets for microRNA-34a-5p in the SLE KEGG pathway (hsa05322) and their structural and functional role in the cell

Gene target in SLE KEGG pathway	Gene symbol	Structural component	Function
**Histone variants**		The core component of the nucleosome which wraps and compacts DNA into chromatin	limiting DNA accessibility to the cellular machinery, thereby play a central role in transcription regulation, DNA replication, and chromosomal stability
Histone H2A type 1-C	HIST1H2AC		
Histone H2A type 1-D	HIST1H2AD		
Histone H2A type 2-A	HIST2H2AA3		
Histone H2AX	H2AFX		
Histone H2A.V	H2AFV		
Histone H2B type 1-D	IST1H2BD		
Histone H2B type 1-J	HIST1H2BJ		
Histone H2B type 1-M	HIST1H2BM		
Histone H2B type 1-O	HIST1H2BO		
Histone H2B type 2-E	HIST2H2BE		
Histone H2B type 3-B	HIST3H2BB		
H3 histone, family 3B	H3F3B		
Histone H4	HIST1H4E		
Histone cluster 1 H2B family member c	HIST1H2BC		
Histone cluster 1 H3 family member j	HIST1H3J		
Histone cluster 1 H4 family member a	HIST1H4A		
Histone cluster 1 H4 family member b	HIST1H4B		
Histone cluster 1 H4 family member d	HIST1H4D		
Histone cluster 1 H4 family member j	HIST1H4J		
Histone cluster 1 H4 family member k	HIST1H4K		
**RNA-binding proteins**			
Small nuclear ribonucleoprotein Sm D3	SNRPD3	The core component of the spliceosomal U1, U2, U4, and U5 small nuclear	The building blocks of the spliceosome involved in the splicing of cellular pre-mRNAs

		ribonucleoproteins (snRNPs)	
Ro60, Y RNA Binding Protein	RO60 or TROVE2	RNA-binding protein	Binds to misfolded non-coding RNAs, pre-5S rRNA, and several small cytoplasmic RNA molecules known as Y RNAs
E3 ubiquitin-protein ligase TRIM21	TRIM21	Forms a ubiquitin ligase complex in cooperation with the E2 UBE2D2 enzymes	mediate ubiquitination of USP4, IKBKB, and CDKN1B
**Immune response**			
T-lymphocyte activation antigen CD86	CD86	Receptor involved in the costimulatory signal essential for T-lymphocyte proliferation and interleukin-2 production, by binding CD28 or CTLA-4	May play a critical role in the early events of T-cell activation and co-stimulation of naive T-cells, such as deciding between immunity and anergy that is made by T- cells within 24 hours after activation
Tumor necrosis factor receptor superfamily member 5	CD40	Receptor for TNFSF5/CD40LG	Transduces TRAF6- and MAP3K8-mediated signals that activate ERK in macrophages and B cells, leading to induction of immunoglobulin secretion
HLA class II histocompatibility antigen, DM alpha chain	HLA-DMA	MHC class II	Plays a critical role in catalyzing the release of class II-associated invariant chain peptide (CLIP) from newly synthesized MHC class II molecules and freeing the peptide binding site for acquisition of antigenic peptides

Table S2. Impact and linkage disequilibrium of MIR34A rs2666433 variant with other SNPs (r^2 >= 0.8) predicted by HaploReg V4.1

**Chr**	**pos (hg38)**	**LD (r^2)**	**LD (D')**	**variant**	**Ref**	**Alt**	**Proteins bound**	**Motifs changed**	**GENCODE genes**
1	9142702	0.86	-0.96	rs34196792	G	A	ZNF 263	BDP1, LUN-1	9kb 3' of MIR34A
1	9147585	0.89	-0.98	rs113390912	C	T		5 altered motifs	4.1kb 3' of MIR34A
1	9153118	1	1	**rs2666433**	A	G		**Ets, PPAR, Pax-4**	1.3kb 5' of MIR34A
1	9154958	0.8	-0.92	rs34174278	A	G		TATA	3.2kb 5' of MIR34A
1	9157629	0.8	-0.92	rs34619897	C	T			5.9kb 5' of MIR34A

Chr: chromosome, pos: position, hg38: human genome release number 38, LD: linkage disequilibrium, Ref: reference allele, Alt: alternative allele. BDP1: B Double Prime 1, a subunit of RNA Polymerase III Transcription Initiation Factor IIIB, LUN-1: a ring finger protein highly expressed in the lung, Ets: "The ETS domain identifies all Ets proteins as sequence-specific DNA-binding proteins", PPAR: Peroxisome-proliferator activated receptor, PAX-4: paired box- 4, TATA: promoter sequences specify the start of the transcription process.
Data source: HaploReg v 4.1. (https://pubs.broadinstitute.org/mammals/haploreg/haploreg.php) (last accessed 27 May 2021).
